# Generation of Broadband High-Purity Dual-Mode OAM Beams Using A Four-Feed Patch Antenna: Theory and Implementation

**DOI:** 10.1038/s41598-019-49377-6

**Published:** 2019-09-10

**Authors:** Yuhan Huang, Xiuping Li, Qingwen Li, Zihang Qi, Hua Zhu, Zaid Akram, Xing Jiang

**Affiliations:** 1 School of Electronic Engineering, Beijing University of Posts and Telecommunications, Beijing, 100876 China; 2 Beijing Key Laboratory of Work Safety Intelligent Monitoring, Beijing University of Posts and Telecommunications, Beijing, 100876 China; 3 Key Laboratory of Universal Wireless Communications, Ministry of Education, Beijing University of Posts and Telecommunications, Beijing, 100876 China; 4 Guangxi College Key Laboratory of Microwave and Optical Wave Applications Technology, Guilin University of Electronic Technology, Guilin, 541004 China

**Keywords:** Electrical and electronic engineering, Electronic and spintronic devices

## Abstract

A broadband four-feed circular patch antenna is demonstrated to generate high-purity dual-mode vortex beams carrying orbital angular momentum (OAM) for the first time. The electric field formula of the circularly polarized (CP) component is derived for analyzing radiation characteristics of different OAM modes, such as the CP property, phase and amplitude distributions. Theoretical calculation and numerical simulation results show that the four-feed patch antenna operating in the TM_*n*1_ mode can generate ±*n* OAM waves. A prototype of a four-feed circular TM_21_-mode patch antenna is fabricated and measured to verify the effectiveness of the theoretical analysis. The proposed antenna consists of a four-feed patch with an air-coupled parasitic patch and a four-way phase-shifting feeding network. Experimental results show that the proposed antenna can generate a left-hand CP OAM with mode *l* = +2 and a right-hand CP OAM with mode *l* = −2. The OAM beams are generated in a wide band from 5.50 to 6.50 GHz with a mode purity over 90%. The proposed method is very suitable for the generation of wideband, dual-mode and high-purity OAM beams in the microwave and millimeter wave bands.

## Introduction

The vortex beam carrying orbital angular momentum (OAM) has received much attention recently for its potential in the field of optical communications^1^, high-capacity communications^2^, the radar field of detection and imaging^3^, etc. The OAM vortex waves feature a helical phase front of ${e}^{-jl\varphi }$, where *l* is the OAM mode order and $\varphi$ is the azimuthal angle. The OAM modes are orthogonal to each other, which enables multiple signals to be transmitted simultaneously at the same frequency. Therefore, OAM can be a useful degree of freedom for increasing the channel capacity and the spectral efficiency^4^. Although the OAM-based radio systems are considered equivalent to conventional multiple-input-multiple-output (MIMO) systems^5^, an experiment demonstrates that OAM-mode division multiplexing systems have low receiver complexity and high capacity compared with conventional line-of-sight MIMO systems^6^. Furthermore, an OAM-based synthetic aperture radar imaging method is experimentally verified, whose azimuth resolution is higher than that of a conventional one^7^. Thus, the potential of OAM in various fields remains to be discussed.

Different methods for generating OAM beams have been reported untill now, yet the generation of broadband and high-purity OAM beams is still considered as a big challenge. Spiral phase plates (SPPs)^8,9^ are widely used in the field of optics due to their simple structures. The SPPs can only generate a single mode of OAM at a time. Also, in the low frequency radio domain, SPPs are of bulky structure which increases the cost. The metallic traveling-wave ring resonant cavity antennas^10,11^ are proposed to generate linearly polarized OAM waves carrying ±3 modes with the purity of 87% and the attenuation coefficient of 0.036^12^. The sizes of these models are large and the bandwidth is quite narrow. Several small-size microstrip antennas have been reported as well, such as the circular patch antenna^13^, the concentric patches antenna^14^, and the half-mode substrate integrated waveguide (HMSIW) antenna^15^. These microstrip antennas are limited to a narrow impedance bandwidth of about 2% and a low OAM purity of about 72%^16^. In addition, the array antennas^17–21^ are proposed to generate OAM beams by controlling a phase shift of the successive element. But the number of the array elements limits the order of the OAM mode and more elements are needed to ensure that the main lobe is in the pure mode region^17^. The OAM purity of 80% with the mode +1 can be achieved at the maximum gain angle by the uniform circular array with 16 elements. Consequently, formerly reported 2 × 2 array antennas^18–21^ can only generate ±1 OAM modes with the main lobe not being in the pure mode region. To increase the channel capacity, a dual linearly polarized (LP) microstrip antenna array is proposed with an impedance bandwidth of 3.6%^18^. A high-profile dual LP bow-tie dipole array antenna achieves a wide bandwidth of 25%^19^. Some dual circularly polarized (CP) patch array antennas^20,21^ are proposed to mitigate the multipath interference, but their feeding networks are quite complex. Several metasurfaces without complex feeding networks are proposed to generate dual-mode OAM beams^22,23^. However, most of these metasurfaces are excited by additional feed horn which makes them difficult to integrate and increase the profile of the antenna. Recently, an innovative OAM generation method based on transformation optic using all-dielectric lens is presented^24^. The lens can generate an OAM mode of +1 from 8 GHz to 16 GHz. In this method, the physical realization of the device is a challenge and radiation patterns of the proposed lens have a slight divergence effect. A phase-modulation converging lens^25^ based on the concept of the optical converging axicon excited by a patch array source can achieve the OAM beam converging from 9.3 GHz to 10 GHz, which is advantageous for improving the propagation performance of the OAM beam.

In this paper, we propose a new strategy to generate broadband high-purity dual-mode OAM beams using the four-feed circular patch antenna. The theoretical formula of the radiation field shows that a four-feed patch antenna at TM_*n*1_ mode can produce ±*n* OAM modes. A prototype of the four-feed circular TM_21_-mode patch antenna is fabricated and measured to verify the effectiveness of the theoretical analysis. The simulated and measured results are well matched with the theoretical ones. The measured 3-dB axial ratio (AR) bandwidth is about 28.33% from 5.00 to 6.70 GHz for both ports. The OAM beams with ±2 modes are generated in a wide band from 5.50 to 6.50 GHz with high mode purity.

## Results

### Theoretical analysis of a four-feed circular patch antenna with CP OAM modes

**Figure 1:**
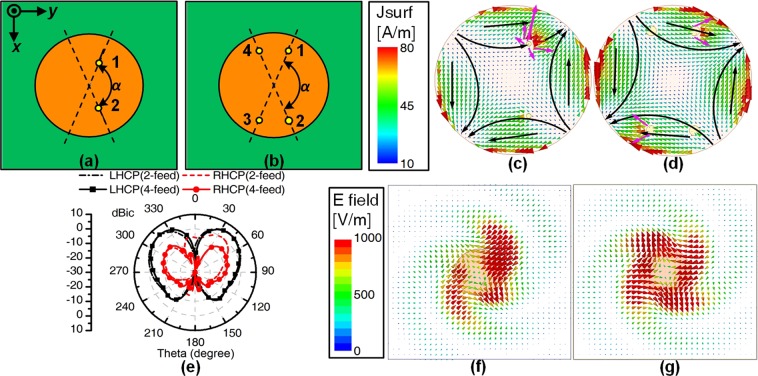
Geometries, current and electric field distributions of the circular patch antennas excited by different feeds. (**a**,**c**,**f**) Two feeds. (**b**,**d**,**g**) Four feeds. (**e**) Simulated normalized radiation patterns. The black arrow represents the direction of the current of the TM_21_ mode. The pink arrow signifies the direction of the clutter current.

The geometries of the circular patch antennas excited by two feeds and four feeds are shown in Fig. 1(a,b). Circular antennas are fed with equal amplitude, a relative 90° phase shift, and the proper angular spacing to obtain CP radiation. In the case of TM_21_-mode patch antenna, the feed angular spacing *α* is set to 135°. We employ two additional feeds 3, 4 located diametrically across from the feeds 1, 2. The four-feed antenna is fed with equal amplitude and a phase shift of $[{0}^{\circ },{90}^{\circ },{0}^{\circ },{90}^{\circ }]$ for the TM_21_ mode. As shown in Fig. 1(d), a more symmetric current distribution is obtained by using four-feed structure. This is because most of the cluttered current due to the mutual coupling between the feeds is suppressed by the two opposing feeds. Moreover, the effect of the remaining cluttered current is significantly eliminated by the opposite current. Thus, the four-feed structure achieves a lower cross-polarization level compared to the two-feed structure, as shown in Fig. 1(e). Additionally, Fig. 1(f,g) compares the electric field distributions for the two-feed antenna and the four-feed antenna. The four-feed structure has a more uniform and symmetrical distribution of the TM_21_ mode because of the low cross polarization. This illustrates that the radiation of the working mode (TM_21_ mode) is enhanced and the fields of the unwanted adjacent modes (TM_11_ and TM_31_ modes) are suppressed. As a result, the CP bandwidth is widened and the mode purity is improved.

Next, a theoretical analysis of a four-feed circular microstrip patch antenna generating CP OAM beams is presented as follows. Based on the cavity-model approximation, different TM_*nm*_ modes can be excited by different circular patch sizes. The symbols *n* and *m* are the number of the angle mode and the radial mode, respectively. The electric field components of the circular patch excited by a single feed in the spherical coordinate are given as follows^26^

1 $$\begin{array}{rcl}{E}_{\theta n}(\varphi ,\theta ) & = & {j}^{n}\frac{{e}^{-j{k}_{0}r}}{2r}{k}_{0}{a}_{e}h{E}_{0}{J}_{n}({a}_{e}{k}_{0}\sqrt{{\varepsilon }_{r}})\,\cos \,n\varphi \,[{J}_{n+1}(\gamma )-{J}_{n-1}(\gamma )]\\ {E}_{\varphi n}(\varphi ,\theta ) & = & {j}^{n}\frac{{e}^{-j{k}_{0}r}}{2r}{k}_{0}{a}_{e}h{E}_{0}{J}_{n}({a}_{e}{k}_{0}\sqrt{{\varepsilon }_{r}})\,\sin \,n\varphi \,[{J}_{n+1}(\gamma )+{J}_{n-1}(\gamma )]\,\cos \,\theta \end{array}$$

where $\gamma ={k}_{0}{a}_{e}\,\sin \,\theta$, *k*_0_ is the phase constant in a vacuum, *a*_*e*_ is the equivalent circular patch radius, *h* is the substrate thickness, *E*_0_ is the electric field amplitude at the edge of the patch, *J*_*n*_ is the Bessel function of the first kind for order integer *n*, and ${\varepsilon }_{r}$ is the relative permittivity. Moreover, the equivalent circular patch radius can be derived by ${a}_{e}={\lambda }_{0}{X}_{nm}/(2\pi \sqrt{{\varepsilon }_{r}})$^27^, where *λ*_0_ denotes the free-space wavelength at the center operating frequency (*f*_0_ = 6 GHz), and *X*_*nm*_ is the *m*th zero of the derivative of *J*_*n*_, which is a constant and independent of frequency. Some values of *X*_*nm*_ are shown in Table 1. Because of the fringe fields, the physical patch radius *a*_*p*_ is slightly smaller than the equivalent one.

**Table 1:** Some Values of *X*_*n*1_.

TM_*n*1_	TM_11_	TM_21_	TM_31_	TM_41_	TM_51_	TM_61_	TM_71_
*X*_*n*1_	1.8412	3.0542	4.2012	5.3176	6.4156	7.5013	8.5578

For the case of four feeds, the excitation amplitudes are same and mutual coupling is neglected. The feed angular spacing is given by $\alpha =(2k+1)\pi /2n,k=0,1,2,\ldots ,n-1$^15^. Additionally, the four-feed antenna has a phase shift of   $[{0}^{\circ },{90}^{\circ },{0}^{\circ },{90}^{\circ }]$ for the even-order modes and  $[{0}^{\circ },{90}^{\circ },{180}^{\circ },{270}^{\circ }]$ for the odd-order modes^27^. For simplicity, only the radiated electric field of the right-hand circular polarization (RHCP) is given. The radiated electric field of the left-hand circular polarization (LHCP) can also be obtained in a similar way. The total electric field components generated by the four orthogonal modes are as follows:

2 $$\begin{array}{rcl}{E}_{\theta n}^{t} & = & {E}_{\theta n}^{1}(\varphi ,\theta )+j{E}_{\theta n}^{2}(\varphi +\alpha ,\theta )+{(-1)}^{n}[{E}_{\theta n}^{3}(\varphi +180^\circ ,\theta )\\  &  & +\,j{E}_{\theta n}^{4}(\varphi +\alpha +180^\circ ,\theta )]\\ {E}_{\varphi n}^{t} & = & {E}_{\varphi n}^{1}(\varphi ,\theta )+j{E}_{\varphi n}^{2}(\varphi +\alpha ,\theta )+{(-1)}^{n}[{E}_{\varphi n}^{3}(\varphi +180^\circ ,\theta )\\  &  & +\,j{E}_{\varphi n}^{4}(\varphi +\alpha +180^\circ ,\theta )]\end{array}$$

where superscripts 1, 2, 3, and 4 correspond to individual electric fields excited by the four feeds. By combining Eqs (1) and (2), the total electric field components are written as

3 $$\begin{array}{rcl}{E}_{\theta n}^{t} & = & {j}^{n}\frac{C}{r}{e}^{-j{k}_{0}r}{e}^{-jn\varphi }[{J}_{n+1}(\gamma )-{J}_{n-1}(\gamma )]\\ {E}_{\varphi n}^{t} & = & {j}^{n+1}\frac{C}{r}{e}^{-j{k}_{0}r}{e}^{-jn\varphi }[{J}_{n+1}(\gamma )+{J}_{n-1}(\gamma )]\,\cos \,\theta \end{array}$$

where *C* is the amplitude constant. Since the vortex EM waves dynamically rotate around the *z*-axis, the electric field components should be obtained in the cylindrical coordinate system.

The $\rho$-, $\varphi$-, and *z*-components of the total electric field in the cylindrical coordinate system are

4 $$\begin{array}{rcl}{E}_{\rho n}^{t} & = & \cos \,\theta \,{E}_{\theta n}^{t}=-\,{j}^{n}\frac{C}{r}{e}^{-j{k}_{0}r}{e}^{-jn\varphi }[{J}_{n-1}(\gamma )-{J}_{n+1}(\gamma )]\,\cos \,\theta \\ {E}_{\varphi n}^{t} & = & {E}_{\varphi n}^{t}={j}^{n+1}\frac{C}{r}{e}^{-j{k}_{0}r}{e}^{-jn\varphi }[{J}_{n-1}(\gamma )+{J}_{n+1}(\gamma )]\,\cos \,\theta \\ {E}_{zn}^{t} & = & -\sin \,\theta \,{E}_{\theta n}^{t}={j}^{n}\frac{C}{r}{e}^{-j{k}_{0}r}{e}^{-jn\varphi }[{J}_{n-1}(\gamma )-{J}_{n+1}(\gamma )]\,\sin \,\theta .\end{array}$$

The radial electric field can be divided into two orthogonal LP components or CP components, which can be expressed as

5 $$\overrightarrow{{E}_{ln}^{t}}={\overrightarrow{e}}_{\rho }{E}_{\rho n}^{t}+{\overrightarrow{e}}_{\varphi }{E}_{\varphi n}^{t}={\overrightarrow{e}}_{L}{E}_{Ln}^{t}+{\overrightarrow{e}}_{R}{E}_{Rn}^{t}$$

where $${E}_{Ln}^{t}=({E}_{\rho n}^{t}-j{E}_{\varphi n}^{t})/\sqrt{2}$$, $${E}_{Rn}^{t}=({E}_{\rho n}^{t}+j{E}_{\varphi n}^{t})/\sqrt{2}$$.

By combining Eqs (4) and (5), the LHCP and RHCP components of the radial electric field are

6 $$\begin{array}{rcl}{E}_{Ln}^{t} & = & 2{j}^{n}\frac{C}{r}{e}^{-j{k}_{0}r}{e}^{-jn\varphi }\,\cos \,\theta \,{J}_{n+1}(\gamma )/\sqrt{2}\\ {E}_{Rn}^{t} & = & -\,2{j}^{n}\frac{C}{r}{e}^{-j{k}_{0}r}{e}^{-jn\varphi }\,\cos \,\theta {J}_{n-1}(\gamma )/\sqrt{2}.\end{array}$$

**Figure 2:**
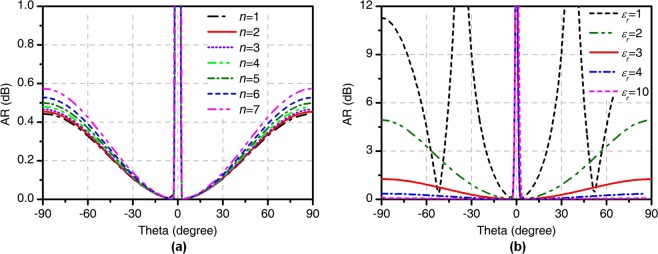
The calculated AR (**a**) for integer orders $n=1,2,3,\ldots ,7$ and (**b**) for $n=2$ with different permittivities.

The calculated AR is defined in decibels as

7 $$AR=20\,{\rm{lg}}|\frac{|{E}_{Ln}^{t}|+|{E}_{Rn}^{t}|}{|{E}_{Ln}^{t}|-|{E}_{Rn}^{t}|}|=20\,{\rm{lg}}|\frac{|{J}_{n-1}(\gamma )|-|{J}_{n+1}(\gamma )|}{|{J}_{n-1}(\gamma )|+|{J}_{n+1}(\gamma )|}|$$

where $\gamma ={k}_{0}{a}_{e}\,\sin \,\theta ={X}_{nm}\,\sin \,\theta /\sqrt{{\varepsilon }_{r}}$. The calculated AR is shown in Fig. 2. For different integer orders with ${\varepsilon }_{r}=3.66$, the AR value is less than 0.6 indicating that the CP radiation is obtained. For the case of the four-feed antenna working in the TM_21_ mode, the AR value decreases with the increase in permittivity of the substrate as shown in Fig. 2(b). Because $|{E}_{Ln}^{t}| < |{E}_{Rn}^{t}|$, the RHCP component is the co-polarized and the LHCP component is the cross-polarized. Equation (6) shows that the RHCP component of the electric field has the helical phase wavefront of ${e}^{-jn\varphi }$, which indicates the generation of the RHCP vortex waves carrying an OAM mode of −*n*. Similarly, the phase wavefront of ${e}^{jn\varphi }$ can be obtained for LHCP, indicating the four-feed antenna can obtain an OAM mode with a positive number.

**Figure 3:**
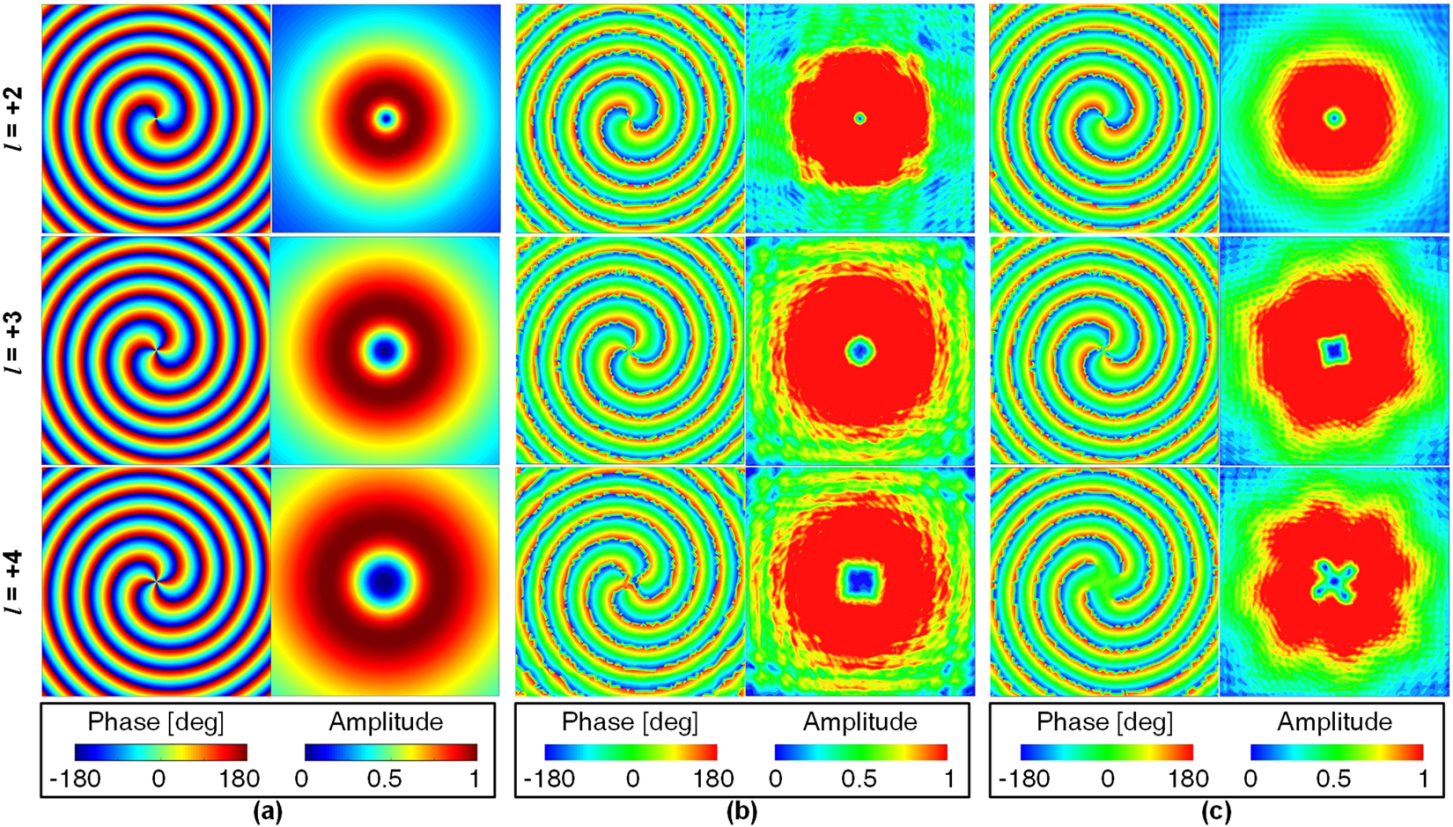
Calculated and simulated phase and amplitude distributions of CP component of the electric field for *l* = +2, *l* = +3, and *l* = +4 OAM modes at 6 GHz. (**a**) Calculated results for the infinite metal ground. (**b**) Simulated results for the metal ground ($10{\lambda }_{0}\times 10{\lambda }_{0}$ square). (**b**) Simulated results for the metal ground ($1.6{\lambda }_{0}\times 1.6{\lambda }_{0}$ square).

To verify the formula of the electric field, the phase and amplitude distributions under ideal conditions operating in the 6 GHz are analysed using MATLAB software, as shown in Fig. 3(a). The square observation window lies at $z=2{\lambda }_{0}$ with a side length of 12*λ*_0_. The phase change along a concentric circle is 2*πl* for *l* = 2, 3, and 4. An amplitude null is observed in the center of the beam, similar to that of the optical vortex beam carrying OAM. Based on the model shown in Fig. 1(b), the theory is further verified by using HFSS software. Figure 3(b,c) shows the simulated results for different OAM modes for different sizes of the metal ground at 6 GHz. The calculated and simulated results for the metal ground of the $10{\lambda }_{0}\times 10{\lambda }_{0}$ square are in good agreement. However, Figure 3(c) reveals a difference in the center of the electric field for the OAM mode of +4 for the metal ground of the $1.6{\lambda }_{0}\times 1.6{\lambda }_{0}$ square, which is mainly due to the effect of the finite metal ground. Both the size of the patch antenna and the divergence angle of the radiation start to rise with an increase in the order of the OAM mode. Therefore, the beam peak of the higher mode antenna is at a lower angle. The finite metal ground cannot maintain the horizontal electric field to propagate well for low-angle radiation, which deteriorates the electric field distribution of the TM_41_ mode. In conclusion, these features with the helical phase and the donut-shaped amplitude validate that the four-feed circular patch antenna can generate ±*n* OAM states for the CP TM_*n*1_ mode.

### Fabricated structure of the antenna working in a CP TM_21_ mode

**Figure 4:**
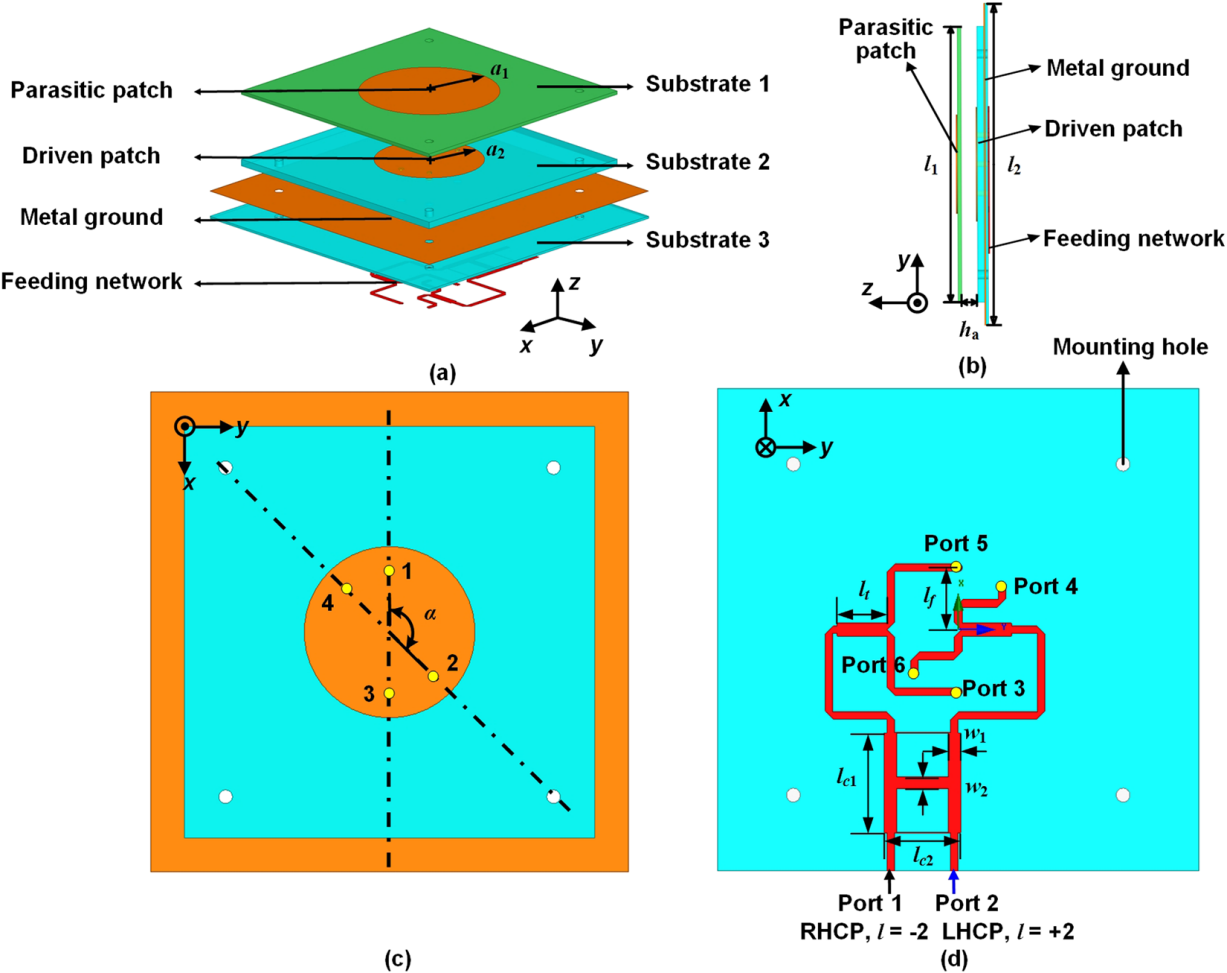
Configuration of the proposed four-feed patch antenna. (**a**) Exploded view. (**b**) Side view. (**c**) Top view without the parasitic patch. (**d**) Bottom view.

The configuration of the proposed antenna consists of a parasitic circular patch, a four-feed circular patch, a metal ground, and a feeding network, as shown in Fig. 4. The parasitic circular patch is located on the top layer of Substrate 1. The four-feed circular patch which works as a driven patch is located on the Substrate 2. There is an air gap between Substrate 1 and Substrate 2 in order to achieve the wideband property of CP radiation. The metal ground with four feeding slots is situated on the top layer of Substrate 3. A four-way feeding network with 90° phase differences is printed on the bottom layer of Substrate 3. An inexpensive FR4 substrate (${h}_{1}=0.8\,{\rm{mm}}$, ${\varepsilon }_{r1}=4.4$, loss tangent = 0.02) is used as the top substrate. The middle and bottom substrates are Rogers 4350B (${h}_{2}=1.524\,{\rm{mm}}$, ${h}_{3}=0.508\,{\rm{mm}}$ ${\varepsilon }_{r2}=3.66$, loss tangent = 0.004). The detailed dimensions of the proposed antenna are set as follows (unit: mm): ${a}_{1}=16$, ${a}_{2}=12.5$, ${l}_{1}=60$, ${l}_{2}=70$, ${h}_{a}=4$, ${l}_{c1}=14.48$, ${l}_{c2}=11.08$, ${w}_{1}=1.78$, ${w}_{2}=1.72$, ${l}_{t}=7.2$, and ${l}_{f}=9$.

**Figure 5:**
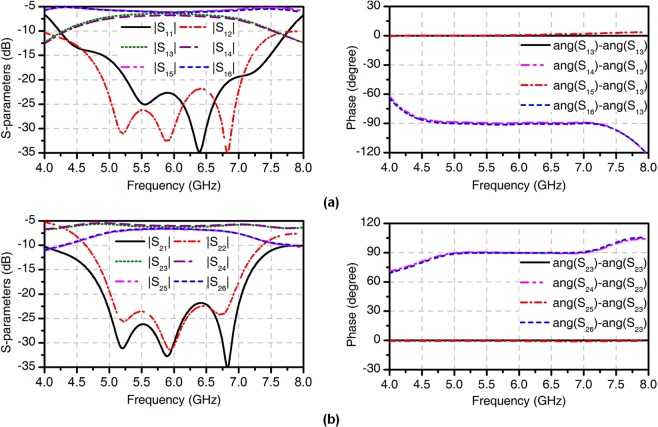
Simulated results of the four-way phase-shifting feeding network. (**a**) *l* = −2 mode. (**b**) *l* = +2 mode.

Figure 4(d) shows the phase-shifting feeding network consisting of a 3-dB three-branch coupler, two T-junction power dividers, and two quarter-wavelength impedance transformers. The 3-dB three-branch coupler is considered as a cascade connection of two conventional couplers^28^, introducing another resonance point, thus providing a broadband 90° phase shift. We designed the feeding network with six ports: Ports 1, 2 are input ports, while other ports are output ports used to feed the patch. When Port 1 is excited and Port 2 is terminated with a 50-Ω load, Ports 3, 4, 5, and 6 have equal amplitude and a phase shift of ${0}^{\circ },-\,{90}^{\circ },{0}^{\circ },-\,{90}^{\circ }$ respectively. The proposed antenna can generate the OAM mode *l* = −2 for RHCP. Whereas when Port 2 is excited and Port 1 is matched, Ports 3, 4, 5, and 6 have equal amplitude and a phase shift of ${0}^{\circ },{90}^{\circ },{0}^{\circ },{90}^{\circ }$ respectively and the OAM mode *l* = +2 is generated for LHCP. The simulated S-parameters of the broadband feeding network are shown in Fig. 5. The simulated $|{S}_{11}|$, $|{S}_{22}|$, $|{S}_{12}|$ and $|{S}_{21}|$ are less than −20 dB from 5.5 to 6.5 GHz. For the OAM mode *l* = −2, the variations of output powers and phases at Ports 3, 4, 5, and 6 are less than 0.9 dB and 0.9° from 5.5 to 6.5 GHz, respectively. For the OAM *l* = +2 mode, the variations of output powers and phases are less than 0.8 dB and 0.5°, respectively. Therefore, the four-way phase-shifting feeding network can maintain a stable phase and amplitude response over a wide frequency band, which makes it possible for the proposed antenna with broadband performance.

### Simulated and measuredt results

**Figure 6:**
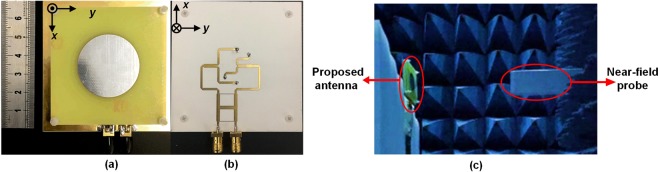
Fabricated antenna. (**a**) Top view. (**b**) Bottom view. (**c**) The near-field test scenario in the anechoic chamber.

**Figure 7:**
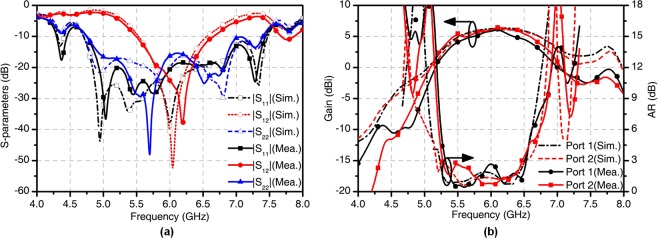
Simulated and measured results of the four-feed antenna. (**a**) S-parameters. (**b**) Gains and AR values versus frequency in *xoz* plane.

**Figure 8:**
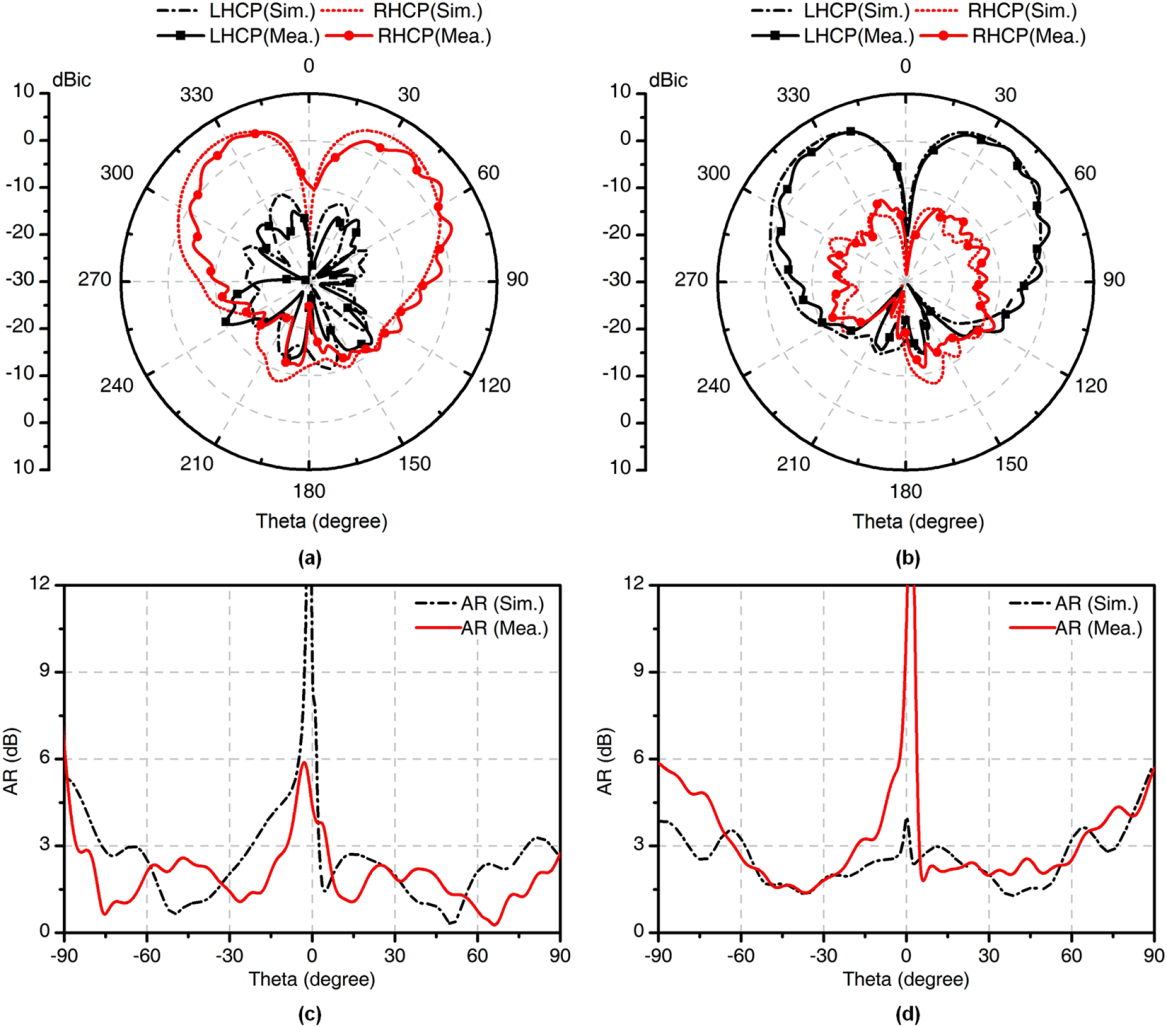
Simulated and measured radiation patterns and AR in *xoz* plane at 6 GHz for (**a**,**c**) *l* = −2 mode, (**b**,**d**) *l* = +2 mode.

**Figure 9:**
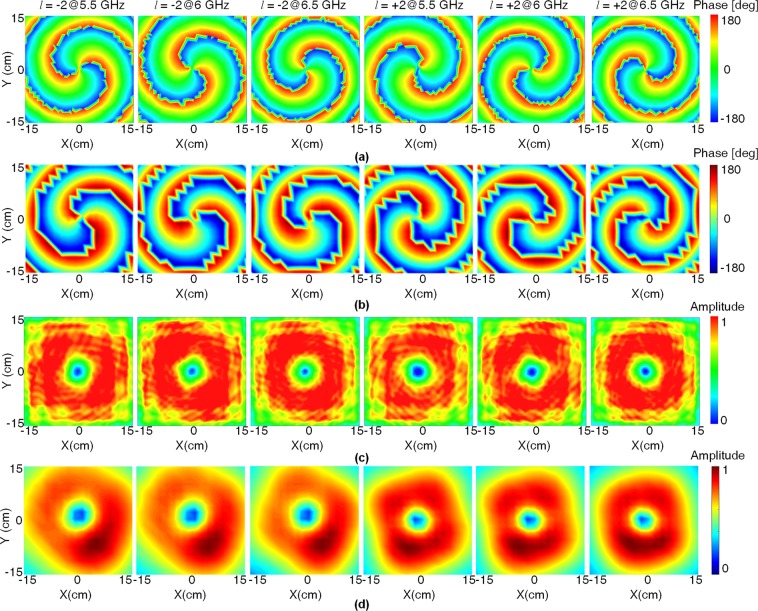
Simulated and measured near-field phase and amplitude distributions of the CP component of the electric field for *l* = −2 and *l* = +2 OAM modes at *z* = 150 mm at different frequencies. (**a**,**c**) Simulated results. (**b**,**d**) Measured results.

To verify the effectiveness of the theory and the design, a prototype of the four-feed antenna is fabricated and measured, as shown in Fig. 6. The simulated and measured S-parameters of the four-feed antenna are shown in Fig. 7(a). The measured 10-dB return loss bandwidth is 45% from 4.75 to 7.45 GHz for Port 1 and 44.2% from 4.75 to 7.40 GHz for Port 2. The measured transmission coefficient $|{S}_{12}|$ is lower than −10 dB from 5.50 to 6.65 GHz. The simulated and measured gains and AR values of the four-feed antenna versus frequency are depicted in Fig. 7(b). The measured 1-dB gain bandwidth of the antenna is 16.67% from 5.50 to 6.65 GHz for both ports. The measured 3-dB AR bandwidth is 28.33% from 5.00 to 6.70 GHz for both ports. The simulated and measured radiation patterns in *xoz* plane at 6 GHz are shown in Fig. 8(a,b). For the OAM with *l* = −2 mode, the angle of the maximum gain is around $\theta =38^\circ$. The measured peak gain is 5.81 dBic at 6 GHz. The half-power bandwidth (HPBW) ranges from −46° to −12° and from 20° to 60°. For the OAM with *l* = +2 mode, the angle of the maximum gain is also around $\theta =38^\circ$ with the peak gain of 6.23 dBic at 6 GHz. The HPBW ranges from −44° to −20° and from 24° to 56°. The measured cross-polarization levels are substantially lower than −16 dB over the HPBW. Figure 8(c,d) shows that the measured AR is substantially below than 3 dB over the HPBW. Figure 9 represents the simulated and measured near-field distributions of the electric field at different frequencies, which highly coincide with each other in a wide band. The observation window lies in the plane at $z=150\,{\rm{mm}}$ with dimensions of 300 mm × 300 mm with a step of 20 mm. The measured and simulated results are in good agreement. The purity of different OAM modes is quantitatively calculated based on the Fourier transform. Since the azimuth angle $\varphi$ is a periodic function, its Fourier conjugate is the OAM spectrum. The Fourier relationship between the OAM spectrum *A*_*l*_ and the sampling phase $\psi (\varphi )$ is given by^29^

8 $${A}_{l}=\frac{1}{2\pi }\,{\int }_{0}^{2\pi }\,\psi (\varphi )d\varphi \,\exp (\,-\,il\varphi ).$$

**Figure 10:**
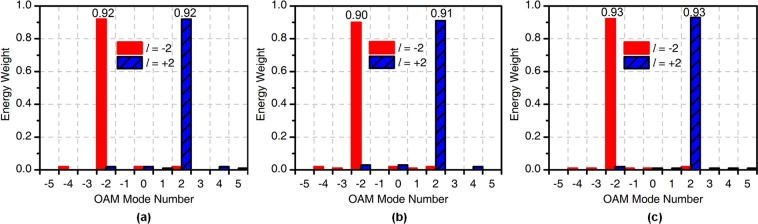
OAM spectrum generated by the four-feed antenna at (**a**) 5.5 GHz, (**b**) 6 GHz, (**c**) 6.5 GHz.

Figure 10 shows the OAM spectrum at maximum gain angle at different frequencies. We can see that the OAM purity of *l* = ±2 is greater than 90% from 5.5 to 6.5 GHz.

## Discussion

In this paper, a four-feed circular patch antenna is proposed for the first time to generate high-purity OAM beams. In principle, arbitrary OAM modes can be obtained by changing the patch radius together with the feed angular spacing. The fabricated prototype of the proposed TM_21_-mode antenna has an ability to generate the OAM mode of *l* = +2 with LHCP and the OAM mode of *l* = −2 with RHCP. Compared with previously reported OAM antennas shown in Table 2, the proposed antenna has a broader bandwidth of about 44.2% and a higher mode purity above 90%. Unlike the designs with single polarization^10,14,15^ and dual LP property^18,19^, the proposed antenna has the benefit of providing dual CP property. Moreover, in comparison with the patch antenna^13^ and array antennas^18–21^, the proposed antenna has a higher order of the OAM mode. All of these attractive features illustrate that the proposed four-feed circular patch antenna offers a tremendous potential in high-capacity wireless communications and radar applications of detection and imaging.

**Table 2:** Comparison Between the Proposed and Reported OAM Antennas.

Ref.	Antenna structure	*f*_0_ (GHz)	Size (${{\boldsymbol{\lambda }}}_{{\bf{0}}}^{{\bf{3}}}$)	10-dB BW	Gain (dBi)	Polarization	OAM Mode	Mode Purity	No. of Ports
^10^	Metallic circular slot + Coupler	10	*π* × 2.06^2^ × N.A	Narrow	3.71	LP	+3/−3	87%	2
^13^	Circular patch + A coupler	2.6	3.4 × 3.4 × 0.12	2.6%	6.5 dBic	Dual CP	+1/−1	>72%	2
^14^	Concentric patches	5.65	*π* × 1.13^2^ × 0.03	Narrow	N.A	CP	+1/−1, +2/−2	72%	6
^15^	HMSIW	10	3 × 3 × 0.07	Narrow	N.A	LP	+2/−2, +6/−6	72%	4
^18^	2 × 2 Patch array + T-junction power dividers	5.5	2.2 × 2 × 0.1	3.6%	9.5	Dual LP	+1/−1	<80%	2
^19^	2 × 2 Dipole array + 4 Couplers	2.4	1.76 × 1.76 × 0.51	25%	N.A	Dual LP	+1/−1	<80%	4
^20^	2 × 2 Patch array + 4 Couplers	4.8	1.67 × 1.67 × 0.1	33.5%	9 dBic	Dual CP	+1/−1	<80%	2
^21^	2 × 2 Patch array + P-i-n didoes feed network	2.5	1.28 × 1.28 × 0.07	21%	5.3	Dual CP	+1/−1	<80%	1
**This work**	**Four-feed circular patch** + **A coupler**	**6**	**1.4** × **1.4** × **0.14**	**44.2%**	**6.2 dBic**	**Dual CP**	+**2/**−**2**	**90%**	**2**

## Methods

The theoretical calculation results are processed by MATLAB software. The proposed four-feed circular patch antenna is designed by ANSYS High Frequency Structure Simulator (HFSS), and manufactured on the Rogers 4350B and FR4 substrates using the PCB technology.

The S-parameters are measured by the Keysight N5247A vector network analyzer and the field distributions are obtained by near-field scanning with an open-ended waveguide probe. The radiation pattern and near-field scanning are measured in a microwave anechoic chamber.

## References

[1] Allen L, Beijersbergen MW, Spreeuw R, Woerdman J (1992). Orbital angular momentum of light and the transformation of Laguerre-Gaussian laser modes. Physical Review A.

[2] Yan Y (2014). High-capacity millimetre-wave communications with orbital angular momentum multiplexing. Nature Communications.

[3] Liu K, Cheng Y, Li X, Jiang Y (2018). Passive OAM-based radar imaging with Single-In-Multiple-Out mode. IEEE Microw. Wirel. Compon. Lett..

[4] Wang J (2012). Terabit free-space data transmission employing orbital angular momentum multiplexing. Nature Photonics.

[5] Oldoni M (2015). Space-division demultiplexing in orbital-angular-momentum-based MIMO radio systems. IEEE Trans. Antennas Propag..

[6] Zhang W (2017). Mode division multiplexing communication using microwave orbital angular momentum: An experimental study. IEEE Trans. Wireless Commun..

[7] Bu X (2018). Implementation of vortex electromagnetic waves high-resolution synthetic aperture radar imaging. IEEE Antennas and Wireless Propagation Letters.

[8] Hui X (2015). Ultralow reflectivity spiral phase plate for generation of millimeter-wave OAM beam. IEEE Antennas Wireless Propag. Lett..

[9] Zhang, L., Zhu, H. & Li, X. A high gain OAM antenna based on splitting spiral phase plate for C band. In *2017 Sixth Asia-Pacific Conf*. *Antennas and Propag*. *(APCAP)*, 1–3 (2017).

[10] Zhang Z, Zheng S, Jin X, Chi H, Zhang X (2017). Generation of plane spiral OAM waves using traveling-wave circular slot antenna. IEEE Antennas Wireless Propag. Lett..

[11] Hui X (2015). Multiplexed millimeter wave communication with dual orbital angular momentum (OAM) mode antennas. Sci. Rep..

[12] Li, Y., Zheng, S., Jin, X., Chi, H. & Zhang, X. Radiation characteristics of the lossy traveling-wave circular antenna. In *2015 Asia-Pacific Microwave Conference (APMC)*, 1–3 (2015).

[13] Bai X (2015). Dual-circularly polarized conical-beam microstrip antenna. IEEE Antennas Wireless Propag. Lett..

[14] Zhang, Z., Xiao, S., Li, Y. & Wang, B.-Z. A circularly polarized multimode patch antenna for the generation of multiple orbital angular momentum modes. *IEEE Antennas Wireless Propag*. *Lett*. **10** (2017).

[15] Chen Y, Zheng S, Chi H, Jin X, Zhang X (2016). Half-mode substrate integrated waveguide antenna for generating multiple orbital angular momentum modes. Electronics Letters.

[16] Huang, Y. *et al*. A broadband multi-OAM-mode four-feed circular patch antenna with high mode purity. In *2018 IEEE Asia-Pacific Conf*. *Antennas and Propag*. *(APCAP)*, 494–495 (2018).

[17] Yuan T, Cheng Y, Wang H, Qin Y (2017). Mode characteristics of vortical radio wave generated by circular phased array: theoretical and experimental results. IEEE Trans. Antennas Propag..

[18] Li H, Kang L, Wei F, Cai Y-M, Yin Y-Z (2017). A low-profile dual-polarized microstrip antenna array for dual-mode OAM applications. IEEE Antennas Wireless Propag. Lett..

[19] Liu B, Cui Y, Li R (2017). A broadband dual-polarized dual-OAM-mode antenna array for OAM communication. IEEE Antennas Wireless Propag. Lett..

[20] Bai X-D (2017). Experimental array for generating dual circularly-polarized dual-mode OAM radio beams. Sci. Rep..

[21] Liu Q (2018). Circular polarization and mode reconfigurable wideband orbital angular momentum patch array antenna. IEEE Trans. Antennas Propag..

[22] Tang S (2018). High-efficiency dual-modes vortex beam generator with polarization-dependent transmission and reflection properties. Sci. Rep..

[23] Qi X (2019). Generating dual-mode dual-polarization OAM based on transmissive metasurface. Sci. Rep..

[24] Feng R (2018). Orbital angular momentum generation method based on transformation electromagnetics. Optics Express.

[25] Meng Y (2018). Phase-modulation based transmitarray convergence lens for vortex wave carrying orbital angular momentum. Optics Express.

[26] Barbuto M, Trotta F, Bilotti F, Toscano A (2014). Circular polarized patch antenna generating orbital angular momentum. Progress In Electromagnetics Research.

[27] Huang J (1984). Circularly polarized conical patterns from circular microstrip antennas. IEEE Trans. Antennas Propag..

[28] Vogel RW (1992). Analysis and design of lumped- and lumped-distributed-element directional couplers for MIC and MMIC applications. IEEE Trans. Microw. Theory Techn..

[29] Yao E, Franke-Arnold S, Courtial J, Barnett S, Padgett M (2006). Fourier relationship between angular position and optical orbital angular momentum. Optics Express.

